# Ethics of Procuring and Using Organs or Tissue from Infants and Newborns for Transplantation, Research, or Commercial Purposes: Protocol for a Bioethics Scoping Review

**DOI:** 10.12688/wellcomeopenres.23235.1

**Published:** 2024-12-05

**Authors:** Maide Barış, Xiu Lim, Melanie T Almonte, David Shaw, Joe Brierley, Sebastian Porsdam Mann, Trung Nguyen, Jerry Menikoff, Dominic Wilkinson, Julian Savulescu, Brian D. Earp

**Affiliations:** 1 Department of Medical History & Ethics, Marmara University, Istanbul, İstanbul, Turkey; 2 Choate Rosemary Hall, Wallingford, Connecticut, USA; 3 Department of Epidemiology and Biostatistics, Imperial College London, London, England, UK; 4 University of Basel, Basel, Basel-Stadt, Switzerland; 5 Maastricht University, Maastricht, Limburg, The Netherlands; 6 University College London, London, England, UK; 7 Great Ormond Street Hospital Children's Charity, London, England, UK; 8 University of Copenhagen, Copenhagen, Capital Region of Denmark, Denmark; 9 National University of Singapore, Singapore, Singapore; 10 University of Oxford, Oxford, England, UK

**Keywords:** infant; organ donation; tissue donation; biomedical ethics; informed consent; transplantation ethics; scoping review

## Abstract

Since the inception of transplantation, it has been crucial to ensure that organ or tissue donations are made with valid informed consent to avoid concerns about coercion or exploitation. This issue is particularly challenging when it comes to infants and younger children, insofar as they are unable to provide consent. Despite their vulnerability, infants' organs and tissues are considered valuable for biomedical purposes due to their size and unique properties. This raises questions about the conditions under which it is permissible to remove and use these body parts for transplantation, research, or commercial purposes. The aim of this protocol is to establish a foundation for a scoping review that will identify, clarify, and categorise the main ethical arguments regarding the permissibility of removing and using organs or tissues from infants. The scoping review will follow the methodology outlined by the Joanna Briggs Institute (JBI), consisting of five stages: (1) identifying the research question, (2) developing the search strategy, (3) setting inclusion criteria, (4) extracting data, and (5) presenting and analysing the results. We will include both published and unpublished materials that explicitly discuss the ethical arguments related to the procurement and use of infant organs or tissues in the biomedical context. The search will cover various databases, including the National Library of Medicine, Web of Science, EBSCO, and others, as well as grey literature sources. Two raters will independently assess the eligibility of articles, and data from eligible studies will be extracted using a standardised form. The extracted data will then be analysed descriptively through qualitative content analysis.

**Background:**

There has been debate about how to respect the rights and interests of organ and tissue donors since the beginning of transplantation practice, given the moral risks involved in procuring parts of their bodies and using them for transplantation or research. A major concern has been to ensure that, at a minimum, donation of organs or other bodily tissues for transplantation or research is done under conditions of valid informed consent, so as to avoid coercion or exploitation among other moral harms. In the case of infants and younger children, however, this concern poses special difficulties insofar as infants and younger children are deemed incapable of providing valid consent. Due to their diminutive size and other distinctive properties, infants’ organs and tissues are seen as valuable for biomedical purposes. Yet, the heightened vulnerability of infants raises questions about when and whether it is ever permissible to remove these body parts or use them in research or for other purposes. The aim of this protocol is to form the basis of a systematic scoping review to identify, clarify, and systematise the main ethical arguments for and against the permissibility of removing and using infant or newborn (hereafter, “infant”) organs or tissues in the biomedical context (i.e. for transplantation, research, or commercial purposes).

**Methods:**

Our scoping review will broadly follow the well-established methodology outlined by the Joanna Briggs Institute ( Peters *et al.*, 2020). We will follow a five-stage review process: (1) identification of the research question, (2) development of the search strategy, (3) inclusion criteria, (4) data extraction, and (5) presentation and analysis of the results. Published and unpublished bibliographic material (including reports, dissertations, book chapters, etc.) will be considered based on the following inclusion criteria: the presence of explicit (bio)ethical arguments or reasons (concept) for or against the procurement and use of organs or tissues from infants, defined as a child from birth until 1 year old (population), in the biomedical domain, including transplantation, research, and commercial development (context). We will search for relevant studies in the National Library of Medicine (including PubMed and MEDLINE), Virtual Health Library, Web of Science, Google Scholar, EBSCO, Google Scholar, PhilPapers, The Bioethics Literature Database (BELIT), EthxWeb as well as grey literature sources (e.g., Google, BASE, OpenGrey, and WorldCat) and the reference lists of key studies to identify studies suitable for inclusion. A three-stage search strategy will be used to determine the eligibility of articles, as recommended by the JBI methodological guidelines. We will exclude sources if (a) the full text is not accessible, (b) the main text is in a language other than English, or (c) the focus is exclusively on scientific, legal, or religious/theological arguments. All articles will be independently assessed for eligibility between two raters (MB & XL); data from eligible articles will be extracted and charted using a standardised data extraction form. The extracted data will be analysed descriptively using basic qualitative content analysis.

**Ethics and dissemination:**

Ethical review is not required as scoping reviews are a form of secondary data analysis that synthesise data from publicly available sources. Our dissemination strategy includes peer review publication, presentation at conferences, and outreach to relevant stakeholders.

**Results:**

The results will be reported according to the PRISMA-ScR guidelines. An overview of the general data from the included studies will be presented in the form of graphs or tables showing the distribution of studies by year or period of publication, country of origin, and key ethical arguments. These results will be accompanied by a narrative summary describing how each included study or article relates to the aims of this review. Research gaps will be identified and limitations of the review will also be highlighted.

**Conclusions:**

A paper summarising the findings from this review will be published in a peer-reviewed journal. In addition, a synthesis of the key findings will be disseminated to biomedical settings (e.g., conferences or workshops, potentially including ones linked to university hospitals) in the UK, USA, Türkiye, and Singapore. They will also be shared with the academic community and policy makers involved in the organ procurement organisations (OPO), which will potentially consider our recommendations in their decision-making processes regarding infant tissue/organ donation practice in these countries.

**Strengths and limitations of this study:**

- The use of a rigorous, well-established methodological framework will ensure the production of a high-quality scoping review that will contribute to the bioethics literature.
- A comprehensive search of disciplinary and cross-disciplinary databases will be undertaken to ensure coverage of all possible sources that meet the inclusion criteria for the review.
- This review will focus exclusively on infant tissue/organ procurement/use in biomedical contexts, providing a comprehensive and reliable source of ethical arguments for future debates on this sensitive topic.
- The review will be limited to articles published in English, which increases the risk of missing relevant sources published in other languages.
- The review will be limited to articles for which the full text is available, which increases the risk of missing relevant sources that otherwise may have been included in the scoping review had the full text been accessible.

## Plain Language Summary

Ever since organ transplantation became possible in the late 1960s, there has been intense debate about how best to ensure that donors are protected from exploitation or coercion — for example, by family members, healthcare providers, or organ recipients — even in cases where the donor seems willing ( Dunstan, 1997). When voluntary consent to the use of one’s organs or other tissues for transplantation or other biomedical purposes is not possible, associated ethical questions become especially acute. Infants and small children are generally not considered capable of giving their own ethically valid consent to organ or tissue donation among other procedures ( American Academy of Pediatrics Committee on Bioethics, 1995). It is therefore important to determine when, whether, and why/how it is ever ethically permissible to procure or use their body parts for transplantation, research, or commercial purposes ( Watts, 2006). Although various arguments have been given both for and against the moral permissibility of such procurement or use (often described as “donation”), attempts to systematically identify and evaluate these arguments have been few and far between ( Sarnaik, 2015; Weiss *et al.*, 2016). The present article outlines a research strategy for conducting a systematic scoping review of the bioethical arguments for and against this practice.

## Consent to organ or tissue removal: background

According to the 1997 Convention for the Protection of Human Rights and Dignity of the Human Being with Regard to the Application of Biology and Medicine (hereafter referred to as the "Oviedo Convention" because the conference was held in Oviedo, Spain), no tissue or organ shall be procured from a person who lacks the capacity to consent. However, this has not been interpreted as implying that all living individuals incapable of providing meaningful consent should be absolutely barred from becoming organ donors, or otherwise having their organs or tissues removed for biomedical purposes. Instead, Article 20/2 of the Oviedo Convention describes a set of conditions that may, when combined with other ethical, legal, or regulatory factors, allow for certain exceptions to be made. These conditions include the unavailability of a compatible donor who has the capacity to consent; the recipient being a sibling of the donor; the donation having the potential to be life-saving for the recipient; and the potential donor not objecting to donation.

How these considerations apply, or should apply, to infants and young children remains controversial. Indeed, the procurement of tissues or organs from infants, whether pre-mortem or post-mortem, has always been controversial. For instance, in 1999, it was revealed that Alder Hey Children's Hospital in Liverpool, UK had stored thousands of organs and body parts, including hearts and brains, from infants and young children after post-mortem examinations. These tissues were used for research and teaching without the parents' knowledge or consent. This incident, known as the 'Alder Hey scandal', led to a public outcry that eventually triggered the events that resulted in the introduction of the Human Tissue Bill (2004) and the repeal of the Human Tissue Act (1961) in the UK ( Ellis, 2004).

The ethics, criteria for, and regulation of obtaining organs or tissues from minors are especially controversial, whether for transplants, research, paediatric biobanking, or commercial use ( Thys *et al.*, 2016; Van Assche *et al.*, 2016). Given, among other issues, the severe shortage of organs and tissues for transplantation in infants due to their size-related characteristics, the need for a clear and appropriately justified ethical framework to govern the procurement or use of tissues and organs from infants is evident ( Caplan, 1987).

The Dead Donor Rule (DDR), a widely used standard in transplantation medicine, is associated with its own ethical issues in this context. DDR permits the recovery of vital organs only if the donor is confirmed dead, either by neurological or circulatory criteria. While DDR allows organ donation from individuals declared brain-dead, applying the rule to premature infants (born before 37 weeks) is challenging due to the lack of consensus on determining 'brain death' in these cases, as noted by the American Academy of Pediatrics ( AAP Committee on Bioethics, 2013). Furthermore, brain death in infants under one year old is rare, which underscores the difficulty in establishing robust criteria that can be universally applied to this population. Given that most infants in need of tissue or organ transplants are awaiting heart transplants, finding suitable donors is a significant challenge. Since almost all organ donors are brain-dead, the criteria for brain death remain critical. The rarity of brain death in young children exacerbates the difficulty of finding appropriate donors, which can impact the availability of organs for transplantation.

Although tissue or organ procurement after circulatory or brain death is possible yet controversial for major organs such as the heart ( Dalle Ave *et al.*, 2016), the absence of suitable recipients for such small organs may limit its practicality and raise the question of whether the procured tissues or organs should be discarded or used for research purposes. This practical issue complicates the already nuanced ethical considerations of organ removal or use in the paediatric population, particularly in premature infants. The issue is significant because nearly 1 in 10 babies is born prematurely ( Ohuma *et al.*, 2023), and complications of preterm birth are a leading cause of infant mortality ( Siffel *et al.*, 2022). In premature babies, brain stem reflexes may not be fully developed, making it impossible for these reflexes to disappear - a necessary condition for diagnosing brain death according to current criteria. Without clear criteria for diagnosing brain death in preterm infants, ethical and legal decisions regarding organ recovery ^1^ from this specific subpopulation of infants are particularly challenging ( Nakagawa *et al.*, 2011).

Until recently, the use of infant organs or tissues has been limited ( Workman *et al.*, 2013). For example, in the decade between 2010 and 2020, between 3 and 21 neonatal (infants from birth to 28 days of age) "donors" were identified annually in the US, representing 0.03% to 0.21% of the total number of transplant organ donors in a given year ( Anderson *et al.*, 2020). However, as of 5 September 2024, there were 106 infants on the waiting list for donation in the US, highlighting the substantial mismatch between the number of actual infant donors and the number of infants waiting to donate; approximately 1 donor for every 5 infants waiting.

The number of neonatal organ donors for purposes of research is even lower, as families with prenatally diagnosed lethal anomalies, or those experiencing neonatal death, are not typically informed about research donation options ( Anderson *et al.*, 2020). Perhaps more important is the fact that clinicians, the individuals actually approaching the families, are not typically informed about potential research options either ( Vileito *et al.*, 2020). There has thus been an ongoing significant disparity between the desire among medical professionals to use infant organs or tissues for transplantation or research and their availability ( Watts, 2006).

In the mid-2010s, however, some prospective parents dealing with diagnoses of lethal anomalies in their foetuses began choosing to carry pregnancies to term, hoping to then donate (i.e., give permission for retrieval and use of) their neonate’s organs after natural death. These parents contacted the International Institute for the Advancement of Medicine (IIAM), an Organ Procurement Organization (OPO) that annually handles over 15,000 referrals and has placed over 14,000 research organs in the past 20 years ( Anderson *et al.*, 2020). Since then, the use of neonatal organs and tissues for research in areas including “diabetes, pulmonary, gastrointestinal, genitourinary and neurological development, rheumatoid arthritis, autism, childhood psychiatric and neurologic disorders, treatment of MRSA infection and paediatric emergency resuscitation” has risen rapidly ( Anderson *et al.*, 2020). Moreover, not only neonatal but also infant organs and tissues are increasingly used in the cosmetics industry for experimental studies investigating their rejuvenating properties ( Cannovo *et al.*, 2020; Oliveira *et al.*, 2018; Vig *et al.*, 2017). Given that the ethical justification for some of these uses may be considered controversial, it is necessary to comprehensively evaluate existing ethical arguments surrounding infant tissue and organ procurement and use for various purposes in the biomedical context. The primary objective of this review is to identify, systematise, and critically evaluate the ethical arguments for and against the procurement and use of tissue and organs from infants (including neonates and newborns up to 1 year of age) in biomedical contexts.

## Ethical arguments

Numerous arguments surrounding the ethics of consent to organ donation in adults and assent for paediatric populations more generally exist in the bioethics literature ( Katz *et al.*, 2016; Prabhu, 2019; Spriggs, 2023). In this scoping review, we hope to lay the groundwork for a comprehensive ethical analysis of arguments that support or oppose such procurement and use.

Our project thus differs from that of, for instance, Weiss *et al.* (2016), who published a scoping review of bioethical arguments surrounding paediatric organ donation after circulatory determination of death (commonly referred to as DCD). In contrast, our scoping review recognises that there are other contexts - apart from a circulatory determination of death - in which paediatric tissue and organ procurement may occur, particularly when considering the case of paediatric biobanks that collect, store, and manage biological samples ^2^ (such as blood, tissue, DNA, and other bodily materials) from children, typically for medical research or clinical purposes ( Casati *et al.*, 2022). Our review will consider tissue and organ procurement from infants who have been declared dead according to any accepted criteria (e.g., brain death), as well as from living, healthy infants where applicable, ^3^ for relevant biomedical purposes, including transplantation, research, and commercial applications (such as in the skin cosmetics industry). Moreover, the use of leftover tissues from diagnostic testing for transplantation, research or commercial purposes will also be considered. However, the review will not consider issues related to the secondary use of such materials. Therefore, issues like the storage of newborn blood spots collected at birth for routine diagnostic screening for genetic diseases are beyond the scope of this review. We will primarily focus on the use of infant tissues or organs for the benefit of others or third parties.

Our project also differs from that of Bluhme, Henckel, and Jorns who produced a scoping review in which they analysed neonatal organ use from a clinical perspective ( Bluhme *et al.*, 2023). While the review by Bluhme and colleagues summarises the available scientific literature on the potential for neonatal organ procurement and analyses published medical cases of neonatal organ transplantation, ethical arguments regarding whether the procedure itself is justifiable in a biomedical context were not commented upon in that review. On the contrary, our scoping review will focus on ethical issues that are distinctive or different in infants, not only in neonates, while the technical or medical issues will be set aside, except where they generate ethical considerations.

A scoping review was determined to be the ideal method for organising the arguments surrounding the ethics of infant organ or tissue procurement for transplantation, research, and commercial purposes for three main reasons. Firstly, the circumscribed nature of the topic made a comprehensive approach to capturing the existing literature involving the identification of all major perspectives a sensible objective. Secondly, the authors aim to pinpoint knowledge gaps within the current body of research, a task well-suited to the broad and exploratory scope of this review method. Thirdly, the issue involves a wide range of stakeholders, including medical practitioners, researchers, commercial entities, and the infants themselves. A scoping review following the guidelines suggested by JBI aligns with all three of these goals.

Moreover, this scoping review will specifically focus on infants, rather than the broader paediatric population, for several reasons. First, infants, especially those born prematurely or with severe conditions, face unique ethical and clinical challenges, such as brain death determination and the impact of premature birth on organ viability, which differ from those of older children. Second, developmental differences in infants affect both their physiological responses to and the ethical considerations relating to organ procurement. A focused review allows for a detailed exploration of these age-specific factors. Third, ethical issues like brain death determination in very young infants are more complex and less standardised, requiring separate attention to address parental consent and family impact effectively. Fourth, guidelines and regulations for infant organ procurement may differ from those for older children, necessitating a thorough examination of policies tailored to this age group. Fifth, unborn foetuses are excluded because organ and tissue procurement tends to occur outside the mother’s body, while biopsies such as may be taken during pregnancy for diagnostic purposes are excluded. Finally, research on infant organ procurement may highlight distinct trends and challenges compared to broader paediatric studies, offering deeper insights and contributing to specialised protocols and ethical guidelines.

Many studies have addressed bioethical issues surrounding the donation/procurement of infant tissues or organs. However, after conducting a search with the descriptors “bioethics,” “ethics,” “infant or neonatal or newborn” and “organ or tissue donation or transplantation” in the Joanna Briggs Institute Evidence-Based Practice Database, Cochrane Library, PubMed, and Scholar Google, no systematic reviews, overviews, or scoping overviews were found in this specific context.

Study objectives include:

- To determine the depth and breadth of the ethical arguments provided for and against the permissibility of retention of organs or tissues from infants for transplantation, research, or commercial purposes.
- To examine the different ethical arguments that apply to retention and use of organs or tissues from infants who are living, versus those who have died.
- To identify which organs or tissues have been the subject of ethical debate in the bioethics literature in terms of their potential use for these purposes (e.g. heart, lung, bone marrow, skin).
- To identify various purposes of infant organ and tissue use in biomedical settings.
- To identify relevant knowledge gaps in the knowledge and flaws in ethical reasoning that can support the development of novel ethical discussions.

## Methods

A scoping review is a type of systematic review, mapping concepts and findings related to the topic of interest using the knowledge synthesis approach (i.e., the method of systematically collecting, analysing and synthesising evidence from multiple studies or sources to develop a comprehensive understanding of a particular topic or issue). This approach aims to consolidate existing knowledge, identify gaps, and provide evidence-based conclusions or recommendations ( Munn *et al.*, 2018). This scoping review uses all types of evidence at various levels and it employs the well-established scoping review methodology, as described by the Joanna Briggs Institute (JBI), consisting of five-stages: (1) determining the research question, (2) search strategy, (3) inclusion criteria, (4) data extraction, and (5) analysis and presentation of the results.

We will search for relevant studies in the National Library of Medicine (including PubMed and MEDLINE), Web of Science, Google Scholar, EBSCO, Virtual Health Library, The Bioethics Literature Database (BELIT), EthxWeb, and PhilPapers. Grey literature sources will be searched as well to identify relevant non-indexed literature. Third, the reference lists of selected reports and articles will be hand searched for additional studies. The results will be reported in accordance with PRISMA-ScR guidelines. ^4^ Study activities including the writing of this protocol will occur between June 2024-January 2025 or until finished. Ethics Review Board approval is not required for this scoping review.

### Stage 1: Determining the research question

We used an iterative process, as recommended by Arksey & O’Malley (2005), to develop the research question, namely: What are the ethical arguments surrounding the use of infant organs or tissues in the biomedical context (i.e. healthcare/transplantation, research or biotechnology-based commercial purposes)?

Moreover, several sub-questions emerged to guide the review, mentioned previously:

- What are the assumptions about the status of the infant that are made in the arguments for permissibility of organ or tissue harvesting from them? (Population)
- What are the possible gaps in the ethical reasoning regarding the infant organ or tissue procurement? (Concept)
- Which organs or tissues have been the subject of ethical debate in the bioethics literature in terms of their potential use for different purposes? (Context)
- What are the main uses of infant organs or tissues in the biomedical settings? (Context)

### Stage 2: Search strategy

The search strategy will aim to locate both published and unpublished studies. A three-step search strategy will be utilised in this review. An initial search will be performed in two electronic databases, the National Library of Medicine (namely PubMed and MEDLINE) and Virtual Health Library to identify articles on the topic. Two reviewers (MB & XL) will independently conduct searches before screening titles, abstracts, and full texts against eligibility criteria. Disagreement in paper inclusion will be resolved by a third reviewer (BDE). Second, a search strategy, including all identified keywords and index terms, will be adapted for each included database and/or information source. We will search for relevant studies in the National Library of Medicine (including PubMed and MEDLINE), Web of Science, Google Scholar, EBSCO, Virtual Health Library, Google Scholar, The Bioethics Literature Database (BELIT), EthxWeb, and PhilPapers. Third, the reference list of identified reports and articles to be, will be hand searched for additional studies. A grey literature search (including but not limited to unpublished research, evaluation reports, guidelines, committee reports theses, organisational reports, and conference proceedings) will be conducted via Google, BASE (Bielefeld Academic Search Engine), OpenGrey and WorldCat, in addition to the abovementioned databases, to identify any non-indexed literature of relevance. Following the search, all identified citations will be collated and uploaded into EndNote and duplicates will be removed. Then, sources will be imported into Covidence.

The search strategy may include variations of following keywords and controlled terms (e.g., MeSH terms in PubMed, Emtree terms in Embase) adapted to the database, including but not limited to:

- Population: Infants (infant OR newborn OR neonat* OR paediatri* OR pediatri* OR minor OR bab*)
- Concept: Ethical arguments (Ethic* OR moral* OR legal* OR regulat* OR theologic* OR religious)
- Context: Organ procurement in biomedicine (Organ OR tissue OR biomaterial OR biological sample) AND (Procur* OR don* OR transplant* OR retriev* OR us*) AND Biomedicine (Biomed* OR biobank* OR biotechno* OR experiment* OR commerc* use OR research)

### Stage 3: Inclusion criteria

Included studies should address bio/ethical arguments/issues (concept) about infant (population) organ or tissue donation/procurement, with a central focus on biomedicine (context). Studies that elaborate the ethics of infant organ and tissue donation/procurement in the context of transplantation, research, or biotechnology-based commercial purposes will be our main focus. Our review will include various types of sources, such as published articles, review articles, original research, opinion papers, editorials, book chapters, thesis chapters, and reports. However, systematic reviews and meta-analyses will be excluded. By excluding systematic reviews and meta-analyses, this scoping review aims to concentrate on capturing original contributions and detailed ethical debates, offering a comprehensive overview of the current landscape of bioethical issues related to infant organ and tissue donation/procurement. This approach ensures a focus on diverse perspectives and nuanced discussions, rather than merely summarising aggregated conclusions from previous reviews.

Studies published between 2010–2024, developed in any year and duration, will be eligible for the review. The starting date of 2010 was determined because there has been a substantial increase in neonatal organ procurement since the mid-2010s, as reported in the literature ( Anderson *et al.*, 2020). Only studies published in English will be included, as English is the common language of those working on this scoping review. Full articles, as well as book chapters and grey literature published in English, will be considered for inclusion using the eligibility criteria:

- Sources that focus on ethical reasoning about organ or tissue donation/procurement from infants will be included (Population).
- Sources that employ ethical, ethico-legal and/or ethico-theological arguments on infant organ or tissue donation/procurement will be included. However sources focused solely on legal and/or theological arguments will be excluded (Concept).
- Sources that discuss infant organ or tissue procurement only from a scientific perspective within the biomedical contexts, such as for transplantation, research, and/or biotechnology-based commercial use, will be excluded (Context).

Once all identified records have been extracted from the databases, they will be imported to the EndNote and duplicates will be removed. The sources then will be exported as an XML file from EndNote and will be uploaded into Covidence for the process of initial screening. Prior to starting the screening process, two members (MB & XL) of the research team will test the inclusion and exclusion criteria in a random sample of 10% of the uploaded sources. Any disagreement will be discussed among the reviewers, and criteria for inclusion and exclusion, if needed, will be clarified accordingly. Once the final inclusion and exclusion criteria are determined, the team will pull another random sample of retrieved articles to screen and test for inter-rater reliability, using the Kappa coefficient. Until a Kappa of at least 0.85 (considered excellent agreement) is reached, the raters will continue to test inter-rater reliability ( McHugh, 2012). Title and abstract screening will be carried out by the reviewers on all uploaded articles. Based on the inclusion and exclusion criteria, the reviewers will group the articles as ‘Yes’, ‘No’ or ‘Maybe’; all ‘Yes’ and ‘Maybe’ articles will then undergo full-text screening. Articles that are selected for inclusion by both reviewers will then be passed to the the second stage, in which full texts will be carefully reviewed for a final determination about on inclusion or exclusion. Any disagreements will be resolved by discussion or, if necessary, by inviting a third reviewer (BDE).

### Stage 4: Data extraction

Data will be extracted from the included sources by two reviewers (MB & XL) using Covidence online software. To advance the objective of this scoping review, as described above, we will use a draft data extraction form that will be modified iteratively and revised as needed during the data extraction process. Table 1 outlines our provisional data extraction plan for this scoping review.

**Table 1.** Data Extraction Plan.

Category	Data to be Extracted
Article information	Author, journal/publication source, year of publication, DOI/URL, publication type (i.e., academic/scientific paper, grey literature, editorial, press release, organisation report, etc.), study/programme location
Aim of the study	What is the main purpose of the study or article? Is it to make an ethical argument, present the views of a relevant party (e.g. the mother of the infant), propose policy changes, etc.?
Argument for infant donation	What are the arguments *for* infant tissue and organ donation identified within the source?
Arguments against infant donation	What are the arguments *against* infant tissue and organ donation identified within the source?
Main argument of the source	How does the source defend its main argument; what is the source’s stance?
Type of the arguments employed in the source	What type of ethical reasoning/arguments used in the source? (i.e. utilitarian, deontological, ethics of care, etc.)
Type of the tissue/organ	What type of tissues and/or organs from infants does the source focus on?
Purpose of the infant donation	What is the main purpose of the retrieval of tissues and organs from infants mentioned in the source (i.e. research, transplantation, etc.)?
Population description	What are the assumptions about the status of the infant that are made in the arguments for/against permissibility of tissue and organ harvesting from them? By “status” we mean the conditions by which the death of the infant was determined, or whether the infant is living.
Other	“Other” denotes the possibility of creating new categories during the data charting process if needed.

Records identifying each included source will be kept in case further review is required. As data are extracted, it may become prudent to add unanticipated information deemed useful to answering the review question. If so, the data table will be updated in an ongoing fashion by the research team. The data to be extracted will be reviewed by one or two members of the team to ensure that all relevant findings are extracted. During this stage, if any disagreements arise between the reviewers (MB & XL), it will be resolved through discussion, or with the assistance of an additional reviewer (BDE). If required, authors of included sources will be contacted to request missing or additional data.

### Stage 5: Data analysis and presentation

The results of the review will be reported according to the PRISMA-ScR guidelines ( Tricco *et al.*, 2018). Analysis of included sources will be presented in the form of graphs, charts or tables showing the distribution of the studies by year or period of publication, countries of origin, and the main ethical arguments. The results presented in graphs or tables will be also accompanied by a narrative summary describing how the results of each included source relate to the objective and research questions of this review. Gaps in the research and possible limitations of this review will also be highlighted.

A paper summarising the findings from this review will be published in a peer-reviewed journal. In addition, a synthesis of the key findings will be disseminated to biomedical settings (e.g., conferences or workshops, potentially including ones linked to university hospitals) in the UK, USA, Türkiye, and Singapore. They will also be shared with the academic community and policy makers involved in the organ procurement organisations (OPO), which will potentially consider our recommendations in their decision-making process regarding infant tissue/organ donation practice in these countries.

## Patients and public involvement

Patients or the public were not involved in the design, conduct, reporting, or dissemination of our research.

## Ethics and dissemination

Ethical approval is not required as this review is a retrospective review of publicly available sources. The review findings will be disseminated via publication in a peer-reviewed journal, symposia and conference presentations. The findings from this review will inform, through the translation of knowledge, organ procurement organisations (OPO), educational institutions developing infant tissue/organ donation training programs for medical professionals, paediatric and neonatal intensive care professionals.

## Patient consent for publication

Not required.

## Data Availability

No data are associated with this article.

## Footnotes

^1^ Preterm birth is the leading cause of neonatal mortality and is associated with long-term physical and neurodevelopmental challenges. Infants born before 32 weeks are particularly at high risk of death and severe disability, which may make them potential candidates for organ or tissue donation/procurement.

^2^ These samples may come from healthy children or those with specific paediatric conditions, such as congenital disorders and rare diseases.

^3^ Although rare, bone marrow donation from infants might be considered under extreme circumstances, especially if the infant is a match for a sibling (as in 'saviour sibling' cases). Moreover, in some very specific cases, tissues such as skin (for instance, the foreskin following infant or newborn circumcision) or other tissues, including certain types of benign (e.g. hemangiomas) or malignant growths (e.g. neuroblastomas) may be removed, stored in biobanks, and potentially used in research or, very rarely, in transplants. Thus, it can be asserted that while living donation from infants is not a common practice and is, or should be, approached with extreme caution due to the medical, ethical, and legal complexities involved, it is not unheard of.

^4^ Wellcome Open Research asks that systematic review protocols be written in accordance with the PRISMA-P guidelines; however, as this article is a protocol for a scoping review, PRISMA-ScR guidelines were determined to be more appropriate by Tricco *et al.* (2018).
