# Supplementary material for: Ethics of Procuring and Using Organs or Tissue from Infants and Newborns for Transplantation, Research, or Commercial Purposes: Protocol for a Bioethics Scoping Review
Source: Wellcome Open Res. 2024 Dec 5;9:717. [Version 1] doi: 10.12688/wellcomeopenres.23235.1 (PMC11747302; doi:10.12688/wellcomeopenres.23235.1)
Supplement: Peer review history

# Reviewer report: Reviewer response for version 1

**DOI:** 10.21956/wellcomeopenres.25595.r116870

**Published:** 2025-01-22

**Authors:** Kristof Van Assche

**Affiliations:** 1 University of Antwerp, Antwerp, Belgium

The proposed scoping review is of major added value and is expected to greatly improve the current understanding of the ethics surrounding infant organ or tissue procurement. The manuscript could be further improved by addressing the following comments:

 1. The background to the proposed scoping review insufficiently distinguishes between organ/tissue removal from living infants and post-mortem organ/tissue removal from infants. For example, the introductory paragraph refers to the Oviedo Convention, which only concerns living donation from persons unable to consent. This is in contrast with the second paragraph, which begins by discussing how these considerations apply but then shifts focus to post-mortem donation (which is not covered by the Oviedo Convention). Since living and post-mortem donation are governed by different legal and ethical frameworks, a reference to the Additional Protocol concerning Transplantation ( https://rm.coe.int/1680081562) and to the 2010 WHO Guiding Principles ( https://iris.who.int/bitstream/handle/10665/341814/WHO-HTP-EHT-CPR-2010.01-eng.pdf) would be welcome. The entire background is focused on post-mortem organ/tissue removal from infants. It is therefore not clear from the background that the scoping paper will also focus on organ/tissue removal from living infants (as becomes clear only later).

 2. The background seems to conflate different scenarios such as organ/tissue removal for transplantation, biobanking (research), post-mortem investigation, and residual biological material (e.g., foreskin). The ethical arguments vary widely depending on the context (e.g., living vs. deceased donors; the type of organ/tissue; the intended purpose). This is only acknowledged later in the study objectives and research questions, but it would be beneficial to highlight this differentiation already in the background.

 3. The background and study objectives give the impression that scenarios such as living tissue/organ donation by neonates or tissue removal from neonates for commercial purposes are plausible. These scenarios are highly unlikely. Living tissue/organ donation by neonates is, to the best of my knowledge, not performed due to the legal and ethical conditions outlined in instruments like the Oviedo Convention. Even for savior siblings only umbilical cord blood is typically used, and bone marrow or kidney donation will only be considered much later in life and under exceptional circumstances. Similarly, tissue removal from infants for commercial purposes is improbable since biobanks (at least in Europe) are generally connected to university hospitals and academic research projects.

 4. The claim that “neonatal and infant organs and tissues are increasingly used in the cosmetics industry for experimental studies investigating their rejuvenating properties” is unsupported. The cited references (Cannovo et al., 2020; Oliveira et al., 2018; Vig et al., 2017) do not discuss the cosmetics industry (experimental use in treating burn wounds is not directly related to the cosmetics industry). As far as I know, there is no credible evidence to support the assertion that neonatal or infant tissues are being increasingly used in cosmetics research.

 5. The draft does not address how the review will proceed if no relevant literature is found for certain scenarios, such as living tissue/organ donation by neonates or tissue removal for commercial purposes. How will the arguments surrounding the ethics of infant organ or tissue procurement for transplantation, research, and commercial purposes be organised if corresponding literature is unavailable for specific contexts?

 6. The study objectives are convincing but would benefit from an intersectional approach: ethical considerations are not only dependent on distinctions between living and deceased donors, organ/tissue type, or intended use, but often depend on the intersection of these contexts (e.g., organ donation after death vs organ donation by living neonates).

 7. The scoping review will result in recommendations but does not explain how these recommendations will be formulated. Similarly, one of the sub-questions refers to “possible gaps in ethical reasoning” but the draft does not explain how such gaps can/will be identified. Recommendations are generally not the primary purpose of a scoping paper, and if they are included, they are generally high-level rather than detailed or prescriptive, which seems to be the case here. It would be helpful to explain the methodology for arriving at these substantive recommendations.

 8. To what extent will the discussions/recommendations also engage with or challenge the balancing of ethical (and human rights) arguments in supranational legal frameworks that delineate acceptable practices? For instance, Council of Europe Recommendation CM/Rec(2016)6 on research on biological materials of human origin, Article 12 ( https://search.coe.int/cm?i=090000168064e8ff) and Oviedo, Article 20 provide guidance based on a balancing of ethical (and human rights) arguments. Addressing these frameworks in the discussions/recommendations could offer valuable insights and strengthen the analysis.

 9. The methodology of the scoping review is sound. It may be beneficial to also include harvest* in the search string.

Is the study design appropriate for the research question?

Yes

Is the rationale for, and objectives of, the study clearly described?

Yes

Are sufficient details of the methods provided to allow replication by others?

Yes

Are the datasets clearly presented in a useable and accessible format?

Not applicable

Reviewer Expertise:

Ethical and legal aspects of transplantation and biobanking; abortion; euthanasia; innovative treatments; patient rights; equitable access to healthcare.

I confirm that I have read this submission and believe that I have an appropriate level of expertise to confirm that it is of an acceptable scientific standard, however I have significant reservations, as outlined above.

# Reviewer report: Reviewer response for version 1

**DOI:** 10.21956/wellcomeopenres.25595.r116866

**Published:** 2025-01-20

**Authors:** Evie Kendal

**Affiliations:** 1 Swinburne University of Technology, Melbourne, Victoria, Australia

This article outlines a proposed scoping review protocol to investigate appropriate consent procedures for obtaining transplant tissues from infants and newborns. It describes why this  question is important in biomedical research and introduces some of the bioethical concerns regarding exploitation and coercion of donors and families in this context. The proposed methods are sound and in line with disciplinary norms and expectations. Data extraction, analysis and presentation methods are well described and replicable.

Is the study design appropriate for the research question?

Yes

Is the rationale for, and objectives of, the study clearly described?

Yes

Are sufficient details of the methods provided to allow replication by others?

Yes

Are the datasets clearly presented in a useable and accessible format?

Not applicable

Reviewer Expertise:

Bioethics, reproductive health, emerging technologies

I confirm that I have read this submission and believe that I have an appropriate level of expertise to confirm that it is of an acceptable scientific standard.

# Reviewer report: Reviewer response for version 1

**DOI:** 10.21956/wellcomeopenres.25595.r114860

**Published:** 2024-12-24

**Authors:** Sanwar Siraj

**Affiliations:** 1 The University of Hong Kong, Hong Kong, Hong Kong

I suggest authors address the following issues: 

  

1. The authors will carry out the screening process of literature with two members (for example, MB & XL) to test the inclusion and exclusion criteria in a random sample of 10% of the uploaded sources. I suggest authors include details about the activities carried out and research team members will be involved in each phase of the literature search strategy, literature analysis, and report on findings.
2. The authors disseminate their findings to potentially including ones linked to university hospitals) in the UK, USA, Türkiye, and Singapore. I suggest authors to offer justifications supporting their arguments about the selection.
3. The authors consider their dissemination strategy to include peer review publication, presentation at conferences, and outreach to relevant stakeholders. What if the authors consider school-going students and colleges and social gathering parties where parents and families present to make them aware of the research findings?

Is the study design appropriate for the research question?

Yes

Is the rationale for, and objectives of, the study clearly described?

Yes

Are sufficient details of the methods provided to allow replication by others?

Yes

Are the datasets clearly presented in a useable and accessible format?

Yes

Reviewer Expertise:

Organ Donation and Transplanttaion, Medical Decison Making and Clinical Ethics, Assited Dying, and Euthanasia

I confirm that I have read this submission and believe that I have an appropriate level of expertise to confirm that it is of an acceptable scientific standard.

# Response

**Published:** 2025-01-01

**Authors:** Maide Barış

**Affiliations:** Medical History & Ethics, Marmara University, Istanbul, İstanbul, Turkey

**Reviewer comment: The authors will carry out the screening process of literature with two members (for example, MB & XL) to test the inclusion and exclusion criteria in a random sample of 10% of the uploaded sources. I suggest authors include details about the activities carried out and research team members will be involved in each phase of the literature search strategy, literature analysis, and report on findings.** **Authors’ response:** We thank the Dr Siraj for raising this point. Here is a preliminary list of the activities of team members in different stages* of the project, to be added to the protocol:

- Maide Barış: Stage-1; Stage-2; Stage-3; Stage-4; Stage-5
- Xiu Lim: Stage-1; Stage-2; Stage-3; Stage-4; Stage-5
- Melanie T Almonte: Stage-3; Stage-4; Stage-5
- David Shaw: Stage-4; Stage-5
- Joe Brierley: Stage-4; Stage-5
- Sebastian Porsdam Mann: Stage-4; Stage-5
- Trung Nguyen: Stage-3; Stage-4; Stage-5
- Jerry Menikoff: Stage-4; Stage-5
- Dominic Wilkinson: Stage-3; Stage-4; Stage-5
- Julian Savulescu: Stage-4; Stage-5
- Brian D. Earp: Stage-1; Stage-2; Stage-3; Stage-4; Stage-5

*Stage 1: Determining the research question; Stage 2: Search strategy; Stage 3: Inclusion criteria; Stage 4: Data extraction; Stage 5: Data analysis and presentation  

**Reviewer comment: The authors disseminate their findings to potentially including ones linked to university hospitals) in the UK, USA, Türkiye, and Singapore. I suggest authors to offer justifications supporting their arguments about the selection.**

**Authors' response: ** We thank Dr Siraj for raising this point. We have chosen the UK, USA, Türkiye and Singapore because the university hospitals to which our authors are affiliated in these countries are easily accessible.  

**Reviewer comment: The authors consider their dissemination strategy to include peer review publication, presentation at conferences, and outreach to relevant stakeholders. What if the authors consider school-going students and colleges and social gathering parties where parents and families present to make them aware of the research findings?**

**Authors’ response:** We thank Dr Siraj for raising this point. Unfortunately, we do not have any share in the grant for school outreach or contact with schools. Nevertheless, given the significance of such an outreach and contact, it is something we will consider for the future work.
